# Sinus Hypoplasia Precedes Sinus Infection in a Porcine Model of Cystic Fibrosis

**DOI:** 10.1002/lary.23392

**Published:** 2012-06-18

**Authors:** Eugene H Chang, Alejandro A Pezzulo, David K Meyerholz, Andrea E Potash, Tanner J Wallen, Leah R Reznikov, Jessica C Sieren, Philip H Karp, Sarah Ernst, Thomas O Moninger, Nicholas D Gansemer, Paul B McCray, David A Stoltz, Michael J Welsh, Joseph Zabner

**Affiliations:** Department of Otolaryngology–Head and Neck Surgery, Roy J. and Lucille A. Carver College of Medicine, University of IowaIowa City, Iowa, U.S.A.; Department of Internal Medicine, Roy J. and Lucille A. Carver College of Medicine, University of IowaIowa City, Iowa, U.S.A.; Department of Pathology, Roy J. and Lucille A. Carver College of Medicine, University of IowaIowa City, Iowa, U.S.A.; Department of Radiology, Roy J. and Lucille A. Carver College of Medicine, University of IowaIowa City, Iowa, U.S.A.; Department of Pediatrics, Roy J. and Lucille A. Carver College of Medicine, University of IowaIowa City, Iowa, U.S.A.; Howard Hughes Medical Institute, Roy J. and Lucille A. Carver College of Medicine, University of IowaIowa City, Iowa, U.S.A.

**Keywords:** Sinus development, sinus disease, cystic fibrosis, animal model

## Abstract

**Objectives/Hypothesis:**

Chronic sinusitis is nearly universal in humans with cystic fibrosis (CF) and is accompanied by sinus hypoplasia (small sinuses). However, whether impaired sinus development is a primary feature of loss of the cystic fibrosis transmembrane conductance regulator (CFTR) or a secondary consequence of chronic infection remains unknown. Our objective was to study the early pathogenesis of sinus disease in CF.

**Study Design:**

Animal/basic science research.

**Methods:**

Sinus development was studied in a porcine CF model.

**Results:**

Porcine sinus epithelia expressed CFTR and exhibited transepithelial anion transport. Disruption of the *CFTR* gene eliminated both. Sinuses of newborn CF pigs were not infected and showed no evidence of inflammation, yet were hypoplastic at birth. Older CF pigs spontaneously developed sinus disease similar to that seen in humans with CF.

**Conclusions:**

These results define a role for CFTR in sinus development and suggest the potential of the CF pig as a genetic model of CF-sinus disease in which to test therapeutic strategies to minimize sinus-related CF morbidity.

## INTRODUCTION

Nearly all individuals with cystic fibrosis (CF) develop chronic sinusitis.^1^, ^2^ CF sinus disease can cause symptoms of headache, facial pain, nasal obstruction, chronic congestion, and nasal discharge.^3^–^6^ In CF sinusitis, the sinuses are filled with thick mucus typically infected with *Staphylococcus aureus* and *Haemophilus influenza* in younger patients, and *Pseudomonas aeruginosa* in older patients.^7^–^10^ Although CF sinus disease can be treated with antibiotics, topical irrigations, and surgery, there is no current therapy that prevents or cures sinus disease.

Sinus anatomy is also altered in CF patients, with ethmoid, maxillary, frontal, and sphenoid sinus hypoplasia or aplasia being exceptionally common.^11^ Whether sinus hypoplasia is a developmental consequence of loss of CFTR in utero or arises secondary to chronic sinusitis in childhood remains unknown.^12^, ^13^ Ethical concerns preclude studying CF sinus pathogenesis in the neonatal period, and only a few case reports are available to suggest when sinus disease might begin.^11^, ^14^–^18^

An animal model replicating human CF sinus development and disease would allow us to investigate the early pathogenesis of CF sinusitis. We targeted the porcine gene encoding CFTR to produce *CFTR*^−/−^, *CFTR*^Δ*F508*/Δ*F508*^, and *CFTR*^−/Δ*F508*^ pigs (hereafter referred to as CF pigs).^19^, ^20^ Many aspects of human CF are recapitulated in CF pigs including abnormalities of the pancreas, lung, intestine, liver, and other organs.^20^ Like humans, pigs are born with ethmoid and maxillary sinuses and develop frontal and sphenoid sinuses after birth.^21^ The CF pig provides a unique opportunity to investigate sinus development and the onset of sinus disease in an animal model from birth. We asked three questions. First, is CFTR expressed in the porcine paranasal sinuses and does a lack of CFTR decrease anion transport in the sinuses like it does in the lower airways? Second, do CF pigs have sinus developmental abnormalities at birth in the absence of infection? And third, do CF pigs spontaneously develop sinus disease similar to humans? The results have implications for understanding the pathogenesis and treatment of CF sinusitis.

## MATERIALS AND METHODS

### Animals

We previously reported generation of *CFTR*^+/−^ and *CFTR*^+/Δ*F508*^ pigs, and production of *CFTR*^−/−^ and *CFTR*^−/Δ*F508*^ pigs.^19^, ^20^ Animals were mated, and progeny were studied. Two different cohorts of animals were used for these studies. 1) Newborn piglets were used for studies of newborn sinus volume, skull volume, and birth weight. Non-CF pigs included *CFTR*^+/−^ (n = 4), *CFTR*^+/+^ (n = 3) genotypes; CF pigs were *CFTR*^−/−^ (n = 8) piglets. Newborn piglets were separated from the sow within 6 hours of birth. 2) Older pigs were used for longitudinal studies of sinus development and disease. Pigs were housed in separate animal enclosure units, clean animal facilities, and limited exposure to typical swine pathogens. Studies of older pigs used both *CFTR*^−/−^ (n = 4), *CFTR*^Δ*F508*/Δ*F508*^ (n = 2) and *CFTR*^−/Δ*F508*^ (n = 3) genotypes. Standard procedures for animal husbandry and anesthesia were used (see Material and Methods in the online supplement). The Institutional Animal Care and Use Committees of the Universities of Iowa and Missouri approved all animal experiments.

### Electrophysiological Measurements in Cultured Epithelia

Epithelial tissues were excised from the ethmoid sinus immediately after animals were euthanized. Cultured epithelia were studied in modified Ussing chambers. Epithelia were bathed on both surfaces with solution containing (mM): 135 NaCl, 2.4 K2HPO4, 0.6 KH2PO4, 1.2 CaCl2, 1.2 MgCl2, 10 dextrose, 5 HEPES (pH = 7.4) at 37°C and gassed with compressed air. Voltage (Vt) was maintained at 0 mV to measure short-circuit current (Isc). Transepithelial electrical conductance (Gt) was measured by intermittently clamping (Vt) to +5 and/or −5 mV.

### CT Scanning (Disease, Volume Analysis)

#### Sinus volume

Computed tomography (CT) DICOM data sets were imported into the Amira visualization software platform (Mercury Computer Systems Inc., Chelmsford, MA) for sinus volume analysis. We then used threshold values of voxels on the CT image to separate measurements of the volume of the skull and paranasal sinus. The ethmoid, maxillary sinuses, and frontal and sphenoid sinuses were further segmented by hand. The ethmoid sinus was defined as the sinus medial to the orbit, superior to the maxillary sinus, and posterior to the turbinates. The maxillary sinus was defined as the enclosed sinus lateral to the ethmoid, inferior to the orbit, and superior to the molars. The frontal sinus was defined as the sinus superior to the orbits and posterior to the turbinate. The sphenoid sinus was defined as the sinus posterior to the ethmoid sinuses.

### Microbiology

Standard microbiologic techniques were used to identify and quantify bacteria present in sinus samples (see Material and Methods in the online supplement). Additional identifications were confirmed by API 20E or API 20NE (bioMérieux, Inc., Durham, NC), Vitek (bioMérieux), or 16S sequencing (University of Iowa Clinical Microbiology Laboratory and Iowa State University Diagnostic Laboratory). Quantification of colony-forming units was performed.

### Statistical Analysis

Data are presented as mean ± standard error. Unpaired *t* test was performed using GraphPad Prism version 5.00 for Mac, (GraphPad Software, San Diego, CA, www.graphpad.com). Differences were considered statistically significant at *P* < .05.

## RESULTS

### CF Sinus Epithelia Showed Normal Histopathology at Birth

**Fig. 1:**
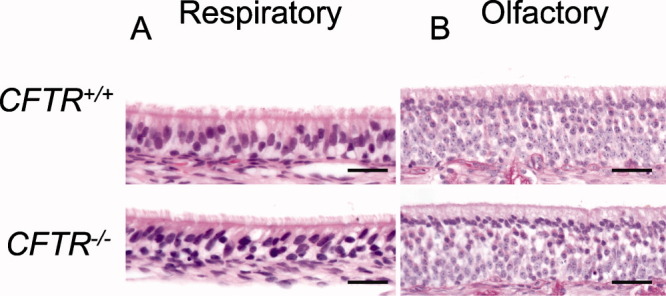
Histopathology of newborn *CFTR*^+/+^ and *CFTR*^−/−^ pig sinus epithelia are similar. Hematoxylin and eosin stains of *CFTR*^+/+^ respiratory (A) and olfactory (B) epithelia show no differences to *CFTR*^−/−^ respiratory and olfactory epithelia in regard to structure, inflammation, or remodeling. Bars: 29 μm.

Our primary goal was to examine CF sinus pathogenesis prior to disease onset. We studied newborn pigs on the first day of life to determine if they had sinus disease at birth. We examined both respiratory (Fig. 1A) and olfactory epithelia (Fig. 1B) of newborn *CFTR*^+/+^ and *CFTR*^−/−^ pigs and could not detect obvious degenerative, inflammatory, or remodeling lesions. We also did not find cellular inflammation, mucus accumulation, goblet cell hyperplasia, or submucosal gland hypertrophy in either group. Consistent with these observations, CT scans and necropsy showed no evidence of sinus epithelial thickening or opacification. In summary, newborn CF pigs had no evidence of sinus infection or inflammation.

### CF Pigs Do Not Express CFTR in the Sinus and Have Anion Transport Defects

**Fig. 2:**
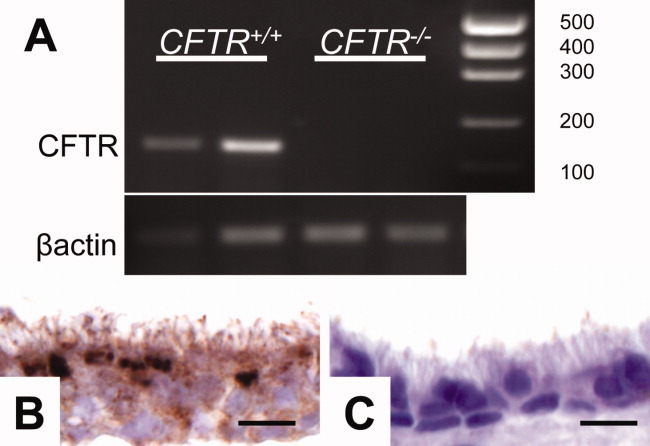
CFTR is expressed in pig sinus epithelia. (A) Reverse-transcriptase polymerase chain reaction of sinus epithelia with primers for *CFTR* (155 bp) and β-actin control. (B) Immunohistochemistry of sinus epithelia staining for CFTR protein in airway epithelia of *CFTR*^+/+^ pigs. (C) Absence of staining in *CFTR*^−/−^ pig airway epithelia. Bars: 8 μm.

Reverse-transcriptase polymerase chain reaction detected CFTR mRNA expression in *CFTR*^+/+^ but not *CFTR*^−/−^ sinus epithelia (Fig. 2A). CFTR protein expression was localized to the apical surface of sinus epithelia in *CFTR*^+/+^ newborn pigs; no expression was detected in *CFTR*^−/−^ sinus epithelia (Fig. 2B,C). To determine if sinus epithelia manifest CFTR anion conductance similar to nasal, tracheal, and bronchial epithelia, we developed well-differentiated *CFTR*^+/+^ and *CFTR*^−/−^ sinus epithelial cell cultures at the air-liquid interface, as previously reported.^22^ We measured both Isc and Gt in Ussing chambers and then altered transport with: 1) amiloride, which inhibits the apical epithelial sodium channel; 2) 4,4′-diisothiocyanotostilbene-2,2′-disulfonic acid (DIDS), which inhibits non-CFTR Cl^−^ channels in the apical membrane; 3) forskolin and 3-isobutyl-2-methylxanthine (IBMX), which increase cellular cyclic adenosine monophosphate (cAMP) levels leading to phosphorylation of CFTR by cAMP-dependent protein kinase; and 4) GlyH-101, which inhibits CFTR channels.^23^

**Fig. 3:**
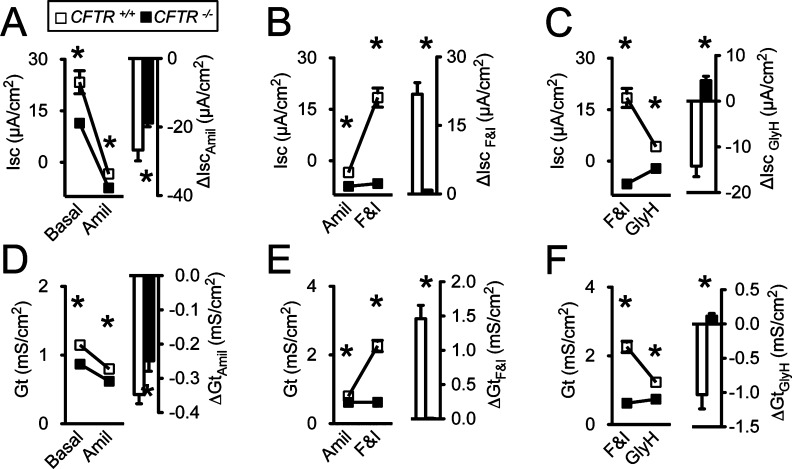
*CFTR*^−/−^ pig sinus epithelia lack Cl^−^ conductance, and have decreased Na+ conductance. Data are presented as mean ± standard error from sinus epithelia cultures of *CFTR*^+/+^ (open symbols and bars) and *CFTR*^−/−^ (closed symbols and bars) pigs. Studies performed on three cultures from four different pigs (n = 4) *P < .05. (A, D) Change in short-circuit current (Isc) and transepithelial electrical conductance (Gt) after adding amiloride (Amil) (100 μM) apically. (B, E) Change in Isc and Gt induced by adding 10 mM forskolin and 100 mM 3-isobutyl-2-methylxanthine (F&I). (C, F) Change in Isc and Gt following addition of GlyH-101 (GlyH) (100 μM).

Both basal Isc and Gt were lower in *CFTR*^−/−^ than *CFTR*^+/+^ sinus epithelia (Fig. 3A,D), a finding consistent with either decreased Na^+^ conductance of CF epithelia, decreased Cl^−^ conductance of CF epithelia, or a combination of both. To investigate Na^+^ conductance we added amiloride to the apical solution; the reductions in Isc (ΔIsc_amil_) and Gt (ΔGt_amil_) were less in CF as compared to non-CF epithelia. This result suggests that the lower basal Isc and Gt in *CFTR*^−/−^ epithelia are partially explained by decreased Na^+^ conductance. Adding DIDS resulted in minimal changes in Isc and Gt in both *CFTR*^+/+^ and *CFTR*^−/−^ sinus epithelia, suggesting non-CFTR Cl^−^ channels are either closed at baseline or play a minimal role in the Cl^−^ conductance of sinus epithelia (data not shown). We assessed CFTR-dependent transport by stimulating with forskolin and IBMX (Fig. 3B,E) and then inhibiting with GlyH-101 (Fig. 3C,F). Forskolin and IBMX increased both Isc and Gt in *CFTR*^+/+^ epithelia, whereas there was no change in *CFTR*^−/−^ epithelia. Additionally, GlyH-101 almost completely inhibited the change in Isc and Gt in the wild-type epithelia. These data are consistent with lack of phosphorylation-dependent activation of CFTR Cl^−^ conductance and transport in *CFTR*^−/−^ sinus epithelia.

### CFTR^−/−^ Pigs Have Sinus Hypoplasia at Birth and Delayed Sinus Development

**Fig. 4:**
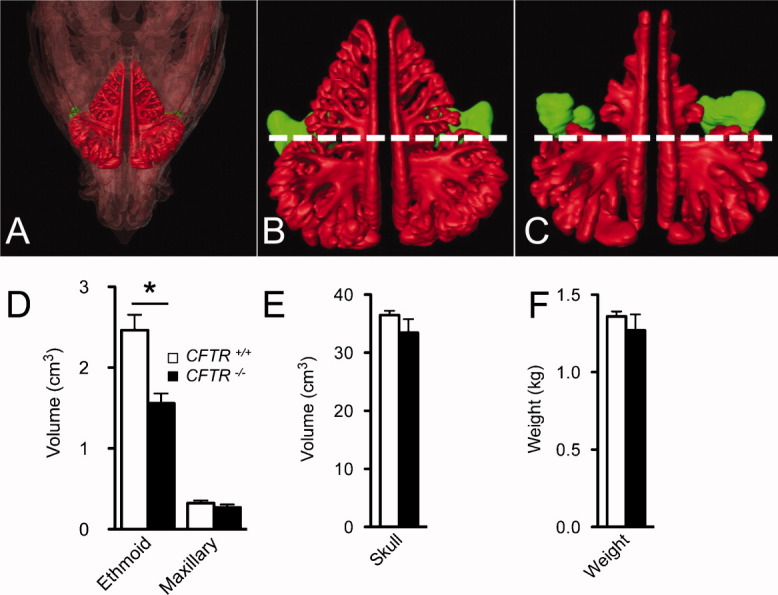
Cystic fibrosis (CF) piglets have ethmoid hypoplasia at birth. (A) Three-dimensional reconstruction view of skull (transparent overlay) and sinus of newborn pig. (B) Non-CF and (C) *CFTR*^−/−^ newborn piglet three-dimensional sinus scan with ethmoid (red) and maxillary (green) sinus. Dotted line dividing anterior and posterior ethmoid sinus. Volumetric analysis performed on three-dimensional reconstructions of segmented sinus computed tomography scans. Data points represent mean ± standard error of non-CF (n = 7; open symbols) and *CFTR*^−/−^ (n = 8; closed symbols) littermate pigs. *P < .05. (C) Volume of ethmoid and maxillary sinuses. (D) Volume of skull. (E) Weight of newborn pigs.

To investigate sinus development, we measured sinus volumes in non-CF and CF pigs by completing three-dimensional reconstructions of skull and sinuses from CT scans of newborn pigs (Fig. 4A). The ethmoid sinuses were hypoplastic and significantly smaller in newborn *CFTR*^−/−^ (n = 8) versus *CFTR*^+/+^ (n = 7) litter-matched piglets (Fig. 4B,C,D). To exclude a calvarial or general growth defect, we measured skull volumes and body weights and found no significant differences between *CFTR*^−/−^ and *CFTR*^+/+^ piglets (Fig. 4E,F). These data suggest that CFTR expression influences prenatal sinus development.

**Fig. 5:**
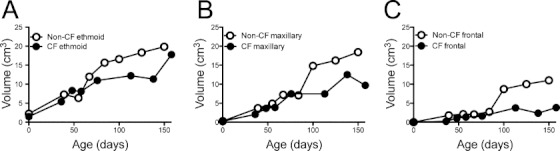
Cystic fibrosis (CF) pigs have sinus hypoplasia and delayed frontal sinus development. Volumetric analysis performed on three-dimensional reconstructions of segmented sinus computed tomography scans. Data points represent mean volumes of one to four non-CF (open symbols) and CF (closed symbols) pigs. Ethmoid (A), maxillary (B), and frontal volumes (C).

Humans with CF have sinus hypoplasia and delayed or absent frontal and sphenoid development.^11^ We hypothesized that CF pigs would also have abnormal sinus development. We investigated the early sinus development in non-CF and CF pigs from birth to 6 months. CF pigs with sinus disease on CT were excluded in order to minimize infection as a confounder. CF pigs had sinus hypoplasia compared to non-CF pigs in the ethmoid and maxillary sinuses (Fig. 5A,B). Additionally, non-CF pigs underwent frontal sinus pneumatization between 3 and 4 months, whereas CF pigs had delayed frontal sinus development (Fig. 5C). The sphenoid sinuses had not developed in either non-CF or CF pigs by 4 months. One confounder was that CF pigs were smaller in size and weight than non-CF pigs, due to malnutrition commonly seen in CF pigs and humans.

### CF Pigs Spontaneously Develop Sinus Disease Similar to Humans

**Fig. 6:**
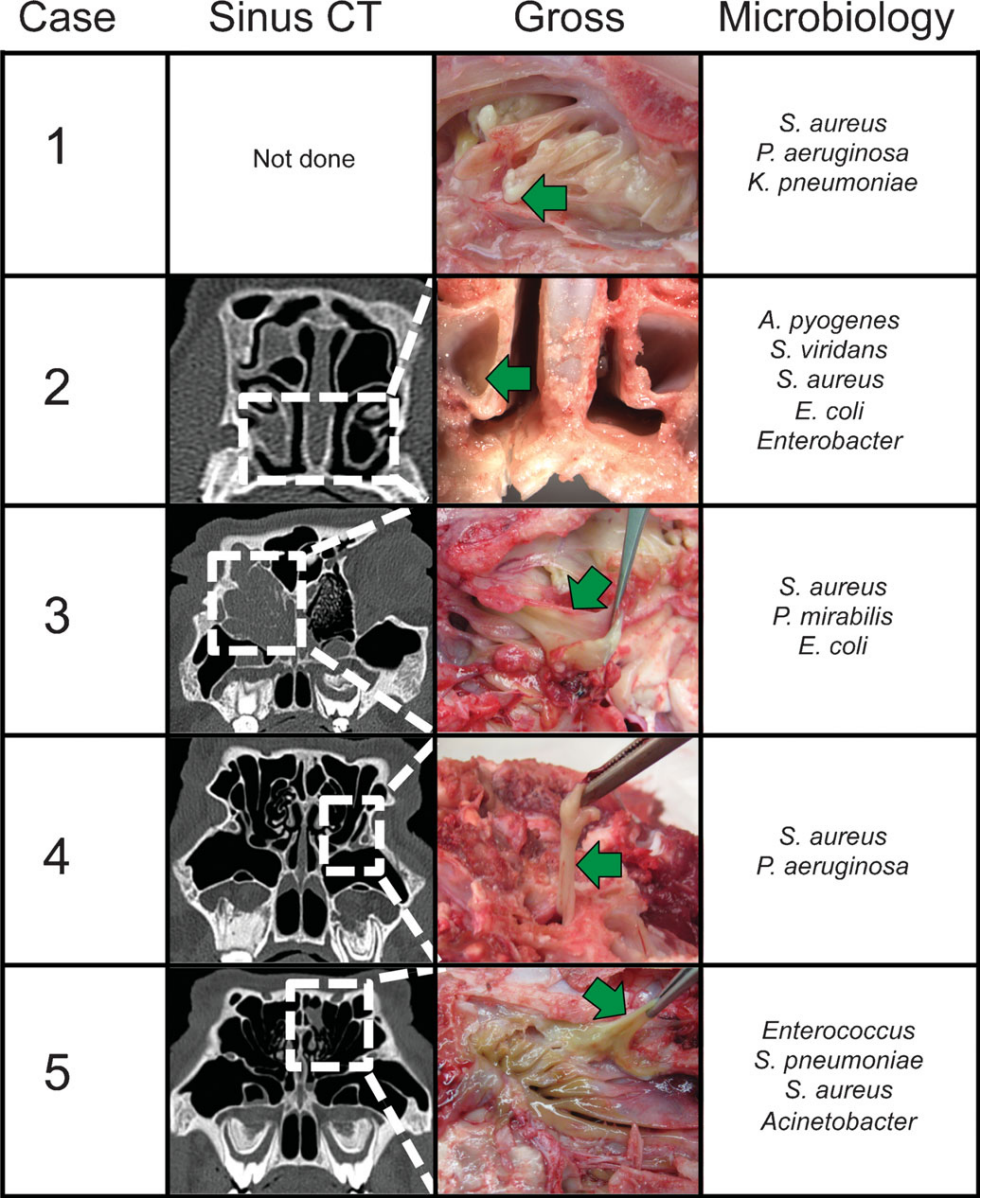
Cystic fibrosis (CF) pigs spontaneously develop sinus disease. Comparison of sinus computed tomography (CT) findings, gross necropsy at death, and microbiology culture results of CF pigs with sinus disease. Dotted white lines on CT highlight the area viewed on gross necropsy. Green arrows highlight thick viscous mucus in the sinuses. Microbiology cultures taken from infected sinus mucus.

Based on these findings and the observation that *CFTR*^−/−^ and *CFTR*^Δ*F508*/Δ*F508*^ pigs develop lung disease, we hypothesized that CF pigs would spontaneously develop sinus disease.^24^, ^25^ Five of nine CF pigs followed longitudinally developed sinus disease confirmed by sinus CT findings, gross disease on necropsy, and/or positive microbiology (Fig. 6).

Case 1 was a *CFTR*^−/−^ pig that developed respiratory stertor and open-mouth breathing at 1 month of age. On necropsy, the pig had extensive mucosal disease in both ethmoid sinuses. Microbiology cultures grew *S. aureus* and *P. aeruginosa*, common pathogens seen in human CF sinus disease.^7^ Case 2 was a *CFTR*^−/−^ pig with right ethmoid and maxillary sinus opacification on sinus CT at 138 days of life. On necropsy, thick mucus occluded the ethmoid and maxillary sinuses. Bacterial cultures grew multiple species including *S. aureus*. Case 3 was a *CFTR*^−/−^ pig that initially showed minimal disease in the right ethmoid sinus on CT at 141 days of age. Later CT scans showed progression of disease that filled the entire right ethmoid sinus and part of the left ethmoid sinus by day 332. Cultures of the thick mucus grew multiple bacteria including *S. aureus*. Case 4 was a *CFTR*^Δ*F508*/Δ*F508*^ pig with minimal sinus disease in the left osteomeatal complex that was detected at 347 days of life. Necropsy 47 days later revealed thick mucus in the left maxillary sinus. Cultures were positive for *S. aureus* and *P. aeruginosa*. Case 5 was a *CFTR*^−/−^ pig that developed left ethmoid cell opacification on CT at 434 days of age. Necropsy revealed thick mucus in the left ethmoid and maxillary sinus. Multiple bacteria including *S*. *aureus* were identified. In summary, several CF pigs developed sinus disease detectable by CT scan, produced thick mucus similar to that seen in human CF, and were infected with bacteria common to human CF sinus and lung disease, including *S. aureus* and *P. aeruginosa*.

**Fig. 7:**
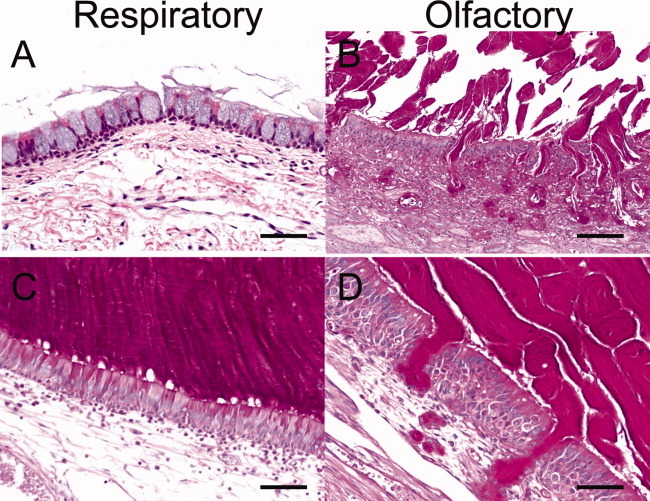
Cystic fibrosis (CF) pig sinus disease shows mucus accumulation and epithelial remodeling on histology. (A, B) Moderate sinus disease in CF pigs showed goblet cell hyperplasia with mucus accumulation and submucosal gland hypertrophy, periodic acid-Schiff (PAS) stain, scale: 50 μm. (C, D) Severe sinus disease with mucus from goblet cells and submucosal glands, PAS stain, bar: 55 μm.

**Fig. 8:**
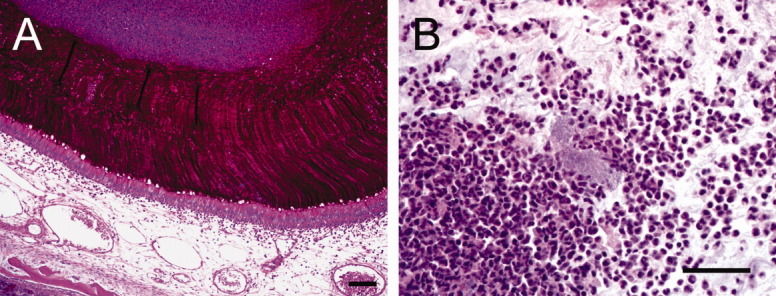
Mucopurulent obstruction of affected sinuses. (A) Lamellar mucus accumulating in the sinus from goblet cells, periodic acid-Schiff stain, bar: 95 μm. (B) Neutrophilic inflammation with bacteria, bar: 40 μm.

On histological examination, CF pigs with sinus disease had hyperplasia of mucus-producing cells, luminal mucus accumulation, and neutrophilic infiltrate. The respiratory epithelia showed goblet cell hyperplasia (Fig. 7A), and the olfactory epithelia exhibited submucosal gland hypertrophy (Fig. 7B). In regions of luminal mucus accumulation, there was a distinct appearance to the mucus. For example, in respiratory epithelia, strands of mucus emanated directly from the goblet cells (Fig. 7C), whereas in the olfactory epithelia the mucus was lamellar and thick and anchored in the submucosal glands, similar to smoke billowing from a chimney (Fig. 7D). In several cases of porcine CF sinus disease, we observed a demarcation between the lamellar mucus overlying the airway and the neutrophilic infiltrate and bacterial infection in the sinus (Fig. 8A,B).

## DISCUSSION

Our data indicate that CF pigs replicate human CF sinus disease with hallmark features of mucus accumulation, inflammation, and bacterial infection. Surprisingly, we found that CF pigs had sinus abnormalities at birth, prior to the onset of infection and inflammation. These observations indicate that CFTR is important for prenatal sinus development and suggest that sinus hypoplasia may contribute to the onset of sinusitis.

### CF pigs have defective anion transport

CF porcine sinus epithelia lack Cl^−^ conductance and exhibit decreased Na^+^ conductance after amiloride. In comparison, Chen et al. have shown that lung epithelia from CF pigs also have markedly decreased Cl^−^ transport and trend toward decreased Na^+^ conductance.^26^ Similar findings have been reported in human CF lung airway epithelia.^27^ These data suggest that CF sinuses do not show Na^+^ hyperabsorption, at least as newborns.

### CFTR regulates sinus development

Sinus hypoplasia is a common finding in CF.^11^ Given the lack of studies in human newborns and infants, it has been assumed that chronic infection leads to the sinus hypoplasia.^12^ Contrary to this belief, our data suggest that CFTR affects sinus development in utero prior to infection or inflammation. The mechanism responsible for reduced sinus development in CF pigs is unknown, although the reduction parallels the reduced tracheal size and airway submucosal gland hypoplasia observed in newborn CF pigs.^28^

There are at least three possibilities for how an ion channel expressed in airway epithelia could affect cartilage and bone development. First, CFTR could globally lower levels of insulin-like growth factor 1 (IGF1), which are decreased in both CF pigs as well as CF infants.^29^ People with Laron syndrome, who are unable to produce IGF1, have sinus hypoplasia and decreased skull size.^30^ However, this possibility seems unlikely because CF pigs have small sinuses yet normal skull volumes. Second, alterations in epithelial liquid secretion could affect sinus development. In both humans and pigs, the ethmoid and maxillary sinuses develop in utero and are fluid-filled at birth. CFTR could play a role in the volume and composition of the liquid that fills the sinuses, thereby altering sinus development. However, the frontal and sphenoid sinuses are commonly hypoplastic in CF, yet they develop after aeration of the sinuses.^31^, ^32^ This suggests that if prenatal liquid transport is important, it cannot be the only cause of sinus hypoplasia. Third, CFTR may play a role in epithelial-mesenychmal interactions, altering cartilage and bone development via locally released growth factors. At birth, CF pigs had smaller ethmoid than maxillary sinuses compared to non-CF littermates. The ethmoid sinus differs from the maxillary sinus as a cartilage template is formed prior to ossification in fetal development (endochondral ossification).^33^ The ethmoid sinus and epithelial-mesenchymal transitions are known to be integral to aspects of craniofacial development.^34^, ^35^ CFTR is likely one of many factors that can regulate prenatal sinus growth.

We observed sinus hypoplasia and delayed frontal sinus development in older CF pigs, similar to changes seen in humans with CF. This finding suggests that CFTR may regulate postnatal sinus growth. Some limitations of this data include the small number of CF pigs followed longitudinally, their relative small size compared to their non-CF controls, and the possibility of concurrent preclinical sinusitis.

### CF pigs develop sinus disease

Sinus disease in five of nine CF pigs was detected by sinus opacification on CT and thick mucus found in necropsy that was culture-positive for common CF pathogens. Interestingly, the four CF pigs that did not develop CF sinusitis were euthanized prior to 4 months of age secondary to either lung disease or systemic illness. Of the five pigs showing CF sinusitis, we were surprised to find that in several cases there was a distinction between the mucus immediately overlying the airway epithelia and the inflammatory and infectious infiltrate above in the sinus. In humans, sinus disease has been described as mucopurulent with the combination of bacteria and neutrophilic inflammation leading to epithelial damage.^36^ In the gut, an organ chronically exposed to ingested bacteria, mucus is critical in preventing bacteria from infiltrating the epithelia and triggering inflammation.^37^ Further studies of goblet cells, submucosal glands, and airway mucus may help answer the question, can mucus be protective by providing a barrier over the airway epithelia?

### The CF pig as a model to test therapeutics

The sinus is easily accessible in the pig and its symmetry allows the opposite sinus to serve as a control.^2^ We can test current therapies including steroids, antibiotics, and surgery, or future therapies such as CFTR potentiators and correctors, and gene therapy to prevent and treat sinusitis. Investigation of the sinuses may also provide insight into CF lung disease. The sinuses and lungs are lined by similar respiratory epithelia, and the sinuses are hypothesized to be a potential reservoir for bacteria, thereby chronically infecting the lungs.^1^, ^7^, ^12^ CF sinus and lung disease also share common disease features such as mucus accumulation, chronic inflammation, and similar pathogenic bacterial infections.^12^, ^38^ We hypothesize that treatment of sinus disease may improve lung disease in the CF pig, a finding that could be applicable in human CF.

## CONCLUSION

CF pigs have sinus hypoplasia at birth and proceed to develop spontaneous sinus disease similar to human CF sinusitis. We speculate that this animal model will aid in the understanding of pathogenesis and be used as a surrogate marker for testing new therapies aimed at preventing CF sinus disease.
