# Supplementary material for: Sinus Hypoplasia Precedes Sinus Infection in a Porcine Model of Cystic Fibrosis
Source: Laryngoscope. 2012 Jun 18;122(9):1898–905. doi: 10.1002/lary.23392 (PMC3449319; doi:10.1002/lary.23392)
Supplement: Supplementary file 1 [file lary0122-1898-SD1.docx]

**Sinus hypoplasia precedes sinus infection in a porcine model of cystic fibrosis**

Eugene H. Chang^1^, Alejandro A. Pezzulo^2^, David K. Meyerholz^3^, Andrea E. Potash^1^, Tanner J. Wallen^1^, Leah R. Reznikov^2^, Jessica C. Sieren^4^, Philip H. Karp^2^, Sarah Ernst^2^, Thomas O. Moninger^2^, Nicholas D. Gansemer^2^, Paul B. McCray, Jr.^5^, David A. Stoltz^2^, Michael J. Welsh^6^, Joseph Zabner^2*^

Departments of Otolaryngology-Head and Neck surgery^1^, Internal Medicine^2^, Pathology^3^, Radiology^4^, Pediatrics^5^, and Howard Hughes Medical Institute^6^,

Roy J. and Lucille A. Carver College of Medicine

University of Iowa, Iowa City, IA

**Online data supplement**

**Methods (Online supplement):**

**Animals**

Pig anesthetic techniques: Newborn piglets were weighed, sedated with ketamine and xylazine. Older pigs were euthanized (Euthasol; Virbac, Fort Worth, TX) if they developed severe systemic disease. Standard procedures for animal husbandry were used. The Institutional Animal Care and Use Committees of the Universities of Iowa and Missouri approved all animal experiments.

**RT-PCR**

The sinus epithelia from the ethmoid and maxillary sinuses was rapidly dissected from the underlying bone and placed into RNA later solution (Ambion). Total RNA was isolated with the Qiagen Lipid Tissue RNeasy kit. RNA quality and concentration were assessed via Nanodrop. RNA was reverse-transcribed with the RT2 First Strand Kit (SA Biosciences). Quantitative RT-PCR was used to measure mRNA. The following primer sequences were used: porcine CFTR (forward, 5′-CTGGAGCCTTCAGAGGGTAAAAT-3′; reverse, 5′-AGTTGGCACGCTTTGATGACACTCC-3′) and actin used as a control (forward, 5′-CTGCGGCATCCACGAAACT-3′; reverse, 5′-GTGATCTCCTTCTGCATCCTGTC-3′). PCR was performed in sample triplicates with RT2 SYBR Green qPCR Master Mixes (SA Biosciences) and a 7500 Fast Real-Time PCR System (Applied Biosystems), following manufacturers’ protocol. Products were separated on a 1.7% agarose gel.

**Immunocytochemistry**

Sinus tissues was excised from newborn piglets, immediately placed in ice-cold 30% sucrose, and quick frozen in OCT. Tissues were cryosectioned at 7 μm thick onto polylysine-coated microscope slides, fixed in 100% MeOH at -20C for 15 min, permeabilized in 0.2% TX-100 (Thermo Scientific) in PBS, and blocked in Super-Block (Thermo Scientific) with 5% normal goat serum (Jackson ImmunoResearch).

**Histopathology and immunohistochemistry**

The sinonasal cavity was dissected, photographed (if required), and placed into 10% neutral buffered formalin for fixation (usually between 48 and 96 h). Sinus tissues were routinely decalcified, processed, embedded, sectioned (4 mm), and stained with hematoxylin and eosin (HE) or amylase pre-treated Periodic acid-Schiff (dPAS) for examination. Whole mount sinus epithelia staining was performed with alcian blue. CFTR immunohistochemistry was performed on decalcified sinus tissues. Briefly, the tissues were sectioned (4 µm) and hydrated through serial alcohol and water baths. Antigen retrieval was performed (citrate buffer pH 6.0, 1000 Watt microwave x 4 minutes and then repeated 3 minutes), endogenous peroxidase activity quenched (3% H2O2, 8 minutes room temperature) and background staining blocked (Fc receptor block, #H9968, 60 minutes, Accurate Chemical and Scientific Corp., Westbury, NY 11590). Primary mouse monoclonal anti-CFTR Ab (1:75, clone M13-4, #MAB3482, Millipore Co., Billerica, MA 01821) was applied followed by rinses (Dako 1X buffer, DAKO, Carpinteria, CA) and a commercial secondary Ab kit (Dako Mouse Envision System, 20 minutes, DAKO, Carpinteria , CA). Commercial chromogen kits were applied (DAKO DAB PLUS - 5 minutes and DAB Enhancer -3 minutes; DAKO, Carpinteria, CA). Sections were counterstained (Surgipath hematoxylin, 1 minute) and then routinely dehydrated (serial alcohols and xylene) and coverslipped.

**Preparation of Differentiated Primary Cultures of Airway Epithelia**

Epithelial cells were isolated from the various tissues by enzymatic digestion, seeded onto permeable filter supports, and grown at the air-liquid interface as previously described ([23](#_ENREF_23)). Differentiated epithelia were used at least 14 days after seeding.

**CT scanning (disease, volume analysis)**

CT data acquisition: volumetric CT data sets were acquired with a 64 SOMATOM Sensation multidetector CT scanner (Siemens Medical Solutions). The following settings were used for scanning: 120-kVp X-ray source voltage, 250 mA/second effective current, and 0.75-mm slice thickness. Animals were sedated with ketamine (20 mg/ kg, intramuscular) and xylazine (1.5 mg/kg, intramuscular) and allowed to breath spontaneously during imaging.

**Microbiology**

Standard microbiologic techniques were used to identify and quantify bacteria present in sinus samples. Samples were serially diluted and plated onto blood agar [tryptic soy agar (TSA) with sheep blood; Remel], Columbia colistin-nalidixic acid agar (Remel), Chocolate agar (Remel), mannitol salt agar (Remel), MacConkey agar (Remel), and Burkholderia cepacia selective agar (Remel). Organisms were identified with standard microbiological procedures.

**Statistical Analysis**

Data are presented as means +/- standard error (SE). Unpaired t test was performed using Graphpad Prism Version 5.00 for Mac, Graphpad Software, San Diego California USA, www.graphpad.com. Differences were considered statistically significant at p < 0.05.
